# Supplementary material for: Psychometric properties of the Adult Primary Care Assessment Tool Short form (PCAT-S) among high-risk patients in Australian general practice
Source: PLoS One. 2026 Feb 6;21(2):e0341250. doi: 10.1371/journal.pone.0341250 (PMC12880635; doi:10.1371/journal.pone.0341250)
Supplement: S1 Table — (DOCX) [file pone.0341250.s001.docx]

**Table S1. Adjustments to the PCAT-S for the EQuIP-GP trial**

| **Subscale** | **Adjustment** |
| --- | --- |
| First contact – Utilization | Item C3 “*When you have to see a specialist, does your PCP have to approve or give you a referral?*” was not included. |
| First contact – Access | No adjustments were made. |
| Ongoing Care | No adjustments were made. |
| Coordination | No adjustments were made. |
| Coordination (Information Systems) | This subscale was not included. |
| Comprehensiveness (Services Available) | This subscale was not included. |
| Comprehensiveness (Services Provided) | No adjustments were made. |
| Family-Centeredness | No adjustments were made. |
| Community Orientation | No adjustments were made. |
| Culturally Competent | No adjustments were made. |

The PCAT-S is an established instrument developed by the Johns Hopkins Primary Care Policy Centre and is freely available for researchers through a request process. The tool can be accessed via this link: <https://publichealth.jhu.edu/johns-hopkins-primary-care-policy-center/primary-care-assessment-tools>.
