# Supplementary material for: Psychometric properties of the Adult Primary Care Assessment Tool Short form (PCAT-S) among high-risk patients in Australian general practice
Source: PLoS One. 2026 Feb 6;21(2):e0341250. doi: 10.1371/journal.pone.0341250 (PMC12880635; doi:10.1371/journal.pone.0341250)
Supplement: S2 Table — The determinant of the correlation matrix that is smaller than 0.00001 suggests an issue with multicollinearity. (DOCX) [file pone.0341250.s002.docx]

**Table S2. Multicollinearity**

The determinant of the correlation matrix that is smaller than 0.00001 suggests an issue with multicollinearity or singularity.

| **Sample** | **Determinant of the correlation matrix** |
| --- | --- |
| No imputation (n = 180) | ${1.4971\times10}^{-5}$ |
| Developer-recommended imputation (n = 373) | ${5.415\times10}^{-4}$ |
| Neutral-value imputation (n = 606) | ${9.002\times10}^{-4}$ |
