# Supplementary material for: Psychometric properties of the Adult Primary Care Assessment Tool Short form (PCAT-S) among high-risk patients in Australian general practice
Source: PLoS One. 2026 Feb 6;21(2):e0341250. doi: 10.1371/journal.pone.0341250 (PMC12880635; doi:10.1371/journal.pone.0341250)
Supplement: S3 Table — Correlations where r>|0.30|) are indicated by an asterisk. (DOCX) [file pone.0341250.s003.docx]

**Table S3. Factor correlations of extracted factors from Exploratory Factor Analysis (EFA) using oblimin rotation, complete observations only (n = 180).** Correlations where $r>\left| 0.30 \right|$) are indicated by an asterisk.

|  | Factor 1 | Factor 2 | Factor 3 | Factor 4 | Factor 5 | Factor 6 | Factor 7 | Factor 8 |
| --- | --- | --- | --- | --- | --- | --- | --- | --- |
| Factor 1 | 1 |  |  |  |  |  |  |  |
| Factor 2 | -0.01 | 1 |  |  |  |  |  |  |
| Factor 3 | 0.06 | 0.21 | 1 |  |  |  |  |  |
| Factor 4 | 0.11 | 0.06 | 0.21 | 1 |  |  |  |  |
| Factor 5 | 0.1 | 0.23 | 0.24 | 0.25 | 1 |  |  |  |
| Factor 6 | 0.04 | 0.11 | 0.37* | 0.2 | 0.32* | 1 |  |  |
| Factor 7 | 0.01 | 0.21 | 0.16 | 0.15 | 0.12 | 0.25 | 1 |  |
| Factor 8 | 0.16 | 0.12 | 0.25 | 0.18 | 0.2 | 0.2 | 0.28 | 1 |
