# Supplementary material for: Psychometric properties of the Adult Primary Care Assessment Tool Short form (PCAT-S) among high-risk patients in Australian general practice
Source: PLoS One. 2026 Feb 6;21(2):e0341250. doi: 10.1371/journal.pone.0341250 (PMC12880635; doi:10.1371/journal.pone.0341250)
Supplement: S6 Table — Confirmatory Factor Analysis (CFA). Problematic items are indicated by an asterisk. Items were considered to load onto a specific factor if the standardised factor loading was > 0.50. Heywood cases occur when the loading is greater than 1.0. Items are presented using wording from the administered survey. (DOCX) [file pone.0341250.s006.docx]

**Table S6. Standardised factor loadings from CFA models by imputation method.**

| **Subscale** | **Item** | **Standardised factor loading** | | |
| --- | --- | --- | --- | --- |
|  |  | **No imputation**  **(n = 180)** | **Developer-recommended imputation**  **(n = 373)** | **Neutral-value imputation**  **(n = 606)** |
| First contact – Utilization | C1 | 0.958 | 0.941 | 0.941 |
|  | C2 | 0.969 | 0.869 | 0.864 |
| First contact – Access | D1 | 0.557 | 0.587 | 0.581 |
|  | D2 | 0.76 | 0.717 | 0.717 |
|  | D3 | 0.593 | 0.565 | 0.469* |
|  | D4 | 0.576 | 0.449* | 0.362* |
| Ongoing Care | E1 | 0.491* | 0.454* | 0.468* |
|  | E2 | 0.71 | 0.651 | 0.66 |
|  | E3 | 0.72 | 0.716 | 0.771 |
|  | E4 | 0.843 | 0.838 | 0.856 |
| Coordination | F1 | 0.708 | 0.643 | 0.706 |
|  | F2 | 0.501 | 0.411* | 0.495* |
|  | F3 | 0.676 | 0.614 | 0.626 |
|  | F4 | 0.836 | 0.858 | 0.847 |
| Comprehensiveness (services provided) | G1 | 0.869 | 0.773 | 0.735 |
|  | G2 | 0.712 | 0.729 | 0.707 |
|  | G3 | 0.744 | 0.672 | 0.662 |
|  | G4 | 0.845 | 0.785 | 0.787 |
|  | G5 | 0.837 | 0.839 | 0.77 |
| Family-Centeredness | H1 | 0.941 | 0.814 | 0.778 |
|  | H2 | 0.751 | 0.613 | 0.636 |
|  | H3 | 0.746 | 0.675 | 0.639 |
| Community Orientation | I1 | 0.703 | 0.669 | 0.511 |
|  | I2 | 0.599 | 0.577 | 0.549 |
|  | I3 | 0.78 | 0.721 | 0.795 |
| Culturally Competent | J1 | 1.191* | 1.242* | 1.129* |
|  | J2 | 0.706 | 0.552 | 0.599 |
|  | J3 | 0.585 | 0.554 | 0.577 |

Confirmatory Factor Analysis (CFA). Problematic items are indicated by an asterisk. Items were considered to load onto a specific factor if the standardised factor loading was >0.50. Heywood cases occur when the loading is greater than 1.0. Items are presented using wording from the administered survey.
