# Supplementary material for: Psychometric properties of the Adult Primary Care Assessment Tool Short form (PCAT-S) among high-risk patients in Australian general practice
Source: PLoS One. 2026 Feb 6;21(2):e0341250. doi: 10.1371/journal.pone.0341250 (PMC12880635; doi:10.1371/journal.pone.0341250)
Supplement: S8 Table — Exploratory factor analysis (EFA) using varimax rotation. The highest loadings for each item are bolded. Items were considered to load onto a specific factor if the factor loading was > 0.40 for that factor and <0.40 for all other factors. Items are presented using wording from the administered survey. (DOCX) [file pone.0341250.s008.docx]

**Table S8. Factor loadings and extracted factors, developer-recommended imputation (n = 373).**

| **Subscale** | **Item** | **Extracted factors** | | | | | | | |
| --- | --- | --- | --- | --- | --- | --- | --- | --- | --- |
|  |  | **1** | **2** | **3** | **4** | **5** | **6** | **7** | **8** |
| First contact – Utilization | C1 | **0.74** | 0.02 | 0.04 | 0 | 0.08 | 0.02 | 0.02 | 0.1 |
|  | C2 | **0.67** | 0.03 | 0.04 | 0.13 | 0.01 | -0.02 | 0 | 0.03 |
| First contact – Access | D1 | 0.21 | **0.42** | 0.1 | 0.02 | 0.09 | 0.17 | 0.06 | -0.02 |
|  | D2 | -0.02 | **0.6** | 0.22 | 0.04 | -0.07 | 0.1 | 0.13 | 0.04 |
|  | D3 | -0.03 | **0.46** | 0.04 | 0.14 | 0.17 | -0.05 | 0.01 | 0.15 |
|  | D4 | -0.02 | **0.52** | -0.06 | 0.03 | 0.18 | -0.06 | -0.05 | 0.05 |
| Ongoing Care | E1 | -0.02 | 0.25 | **0.4** | 0.04 | 0.01 | 0.04 | 0 | 0.01 |
|  | E2 | 0.01 | **0.6** | 0.34 | 0.08 | 0.05 | 0.11 | 0.07 | -0.07 |
|  | E3 | 0.05 | 0.19 | **0.56** | 0.03 | 0.14 | 0.13 | 0.12 | 0.06 |
|  | E4 | 0.05 | 0.12 | **0.69** | 0.09 | 0.18 | 0.16 | 0.05 | 0.1 |
| Coordination | F1 | 0.16 | 0.08 | 0.12 | **0.49** | 0.17 | 0.1 | 0.05 | 0.07 |
|  | F2 | 0 | 0.11 | -0.08 | **0.45** | 0.31 | -0.09 | 0.07 | -0.02 |
|  | F3 | 0.02 | 0.05 | 0.11 | **0.55** | 0 | 0.15 | 0.03 | -0.02 |
|  | F4 | 0.06 | 0.02 | 0.29 | **0.37** | 0.29 | 0.2 | 0.01 | 0.13 |
| Comprehensiveness (services provided) | G1 | 0.05 | 0.04 | 0.15 | 0.04 | **0.65** | 0.34 | 0.05 | 0.11 |
|  | G2 | 0.07 | 0.24 | 0.1 | 0.14 | **0.65** | -0.04 | 0.08 | -0.04 |
|  | G3 | 0.02 | 0.19 | 0.13 | 0.14 | **0.56** | -0.01 | 0.12 | -0.06 |
|  | G4 | 0.04 | 0.03 | 0.23 | 0.13 | **0.58** | 0.32 | 0 | 0.07 |
|  | G5 | 0.02 | -0.05 | **0.44** | 0.15 | 0.33 | 0.23 | 0.12 | 0.21 |
| Family-centeredness | H1 | -0.06 | 0.03 | 0.35 | 0.22 | 0.26 | **0.43** | 0.27 | 0.03 |
|  | H2 | -0.02 | 0.05 | 0.1 | 0.19 | 0.21 | **0.45** | 0.07 | 0.15 |
|  | H3 | 0.04 | 0.18 | 0.25 | 0.06 | 0.02 | **0.57** | 0.21 | 0.11 |
| Community Orientation | I1 | 0.11 | **0.35** | 0.19 | -0.06 | 0.12 | 0.14 | **0.35** | -0.04 |
|  | I2 | 0 | 0.05 | 0.05 | 0.04 | 0.11 | 0.02 | **0.64** | 0.13 |
|  | I3 | 0 | 0.03 | 0.1 | 0.12 | 0.04 | 0.2 | **0.65** | 0.17 |
| Culturally Competent | J1 | 0.11 | 0.09 | **0.59** | 0.14 | 0.14 | 0.06 | 0.14 | 0.37 |
|  | J2 | 0.08 | 0 | 0.14 | 0.01 | 0.08 | 0.05 | 0.1 | **0.7** |
|  | J3 | 0.06 | 0.13 | 0.12 | 0.03 | -0.09 | 0.17 | 0.2 | **0.49** |

Exploratory factor analysis (EFA) using varimax rotation. The highest loadings for each item are bolded. Items were considered to load onto a specific factor if the factor loading was >0.40 for that factor and <0.40 for all other factors. Items are presented using wording from the administered survey.
