# Supplementary material for: Psychometric properties of the Adult Primary Care Assessment Tool Short form (PCAT-S) among high-risk patients in Australian general practice
Source: PLoS One. 2026 Feb 6;21(2):e0341250. doi: 10.1371/journal.pone.0341250 (PMC12880635; doi:10.1371/journal.pone.0341250)
Supplement: S9 Table — Exploratory factor analysis (EFA) using varimax rotation. The highest loadings for each item are bolded. Items were considered to load onto a specific factor if the factor loading was > 0.40 for that factor and <0.40 for all other factors. Items are presented using wording from the administered survey. (DOCX) [file pone.0341250.s009.docx]

**Table S9. Factor loadings and extracted factors using neutral-value imputation (n = 606).**

| **Subscale** | **Items** | **Extracted factors** | | | | | | | |
| --- | --- | --- | --- | --- | --- | --- | --- | --- | --- |
|  |  | **1** | **2** | **3** | **4** | **5** | **6** | **7** | **8** |
| First contact – Utilization | C1 | **0.72** | 0.02 | 0.01 | 0.05 | 0.08 | 0.03 | 0.02 | 0.1 |
|  | C2 | **0.7** | 0 | 0.08 | 0.13 | -0.01 | 0.03 | -0.03 | 0.05 |
| First contact – Access | D1 | 0.18 | **0.45** | 0.1 | 0.06 | 0.17 | -0.03 | 0.12 | -0.07 |
|  | D2 | 0.04 | **0.58** | 0.25 | 0.06 | 0.01 | 0.01 | 0.16 | 0.04 |
|  | D3 | -0.04 | **0.38** | 0.02 | 0.04 | 0.08 | 0.13 | 0.07 | 0.06 |
|  | D4 | -0.06 | **0.49** | -0.01 | 0.04 | -0.01 | 0.17 | -0.09 | 0.06 |
| Ongoing Care | E1 | 0.01 | 0.28 | **0.39** | 0 | 0.02 | 0.09 | 0.03 | 0.06 |
|  | E2 | -0.02 | **0.57** | 0.36 | 0.1 | 0.03 | 0.11 | 0.1 | 0.04 |
|  | E3 | 0.05 | 0.13 | **0.59** | 0.06 | 0.25 | 0.13 | 0.16 | 0.05 |
|  | E4 | 0.08 | 0.14 | **0.66** | 0.08 | 0.29 | 0.08 | 0.08 | 0.15 |
| Coordination | F1 | 0.09 | 0.09 | 0.12 | **0.63** | 0.13 | 0.13 | 0.11 | 0.09 |
|  | F2 | 0.02 | 0.13 | -0.02 | **0.37** | 0.08 | 0.36 | 0.01 | 0.09 |
|  | F3 | 0.09 | 0.03 | 0 | **0.59** | 0.11 | 0.03 | 0.08 | -0.01 |
|  | F4 | 0.03 | 0.15 | 0.22 | 0.39 | **0.42** | 0.04 | 0.01 | 0.09 |
| Comprehensiveness (services provided) | G1 | 0.05 | 0.09 | 0.08 | 0.07 | **0.56** | 0.34 | 0.12 | 0.1 |
|  | G2 | 0.06 | 0.22 | 0.09 | 0.11 | 0.2 | **0.68** | 0.05 | 0 |
|  | G3 | 0.01 | 0.12 | 0.19 | 0.07 | 0.18 | **0.61** | 0.13 | -0.05 |
|  | G4 | 0.07 | 0.1 | 0.09 | 0.08 | **0.73** | 0.24 | 0.05 | 0.05 |
|  | G5 | 0.02 | 0.07 | 0.25 | 0.14 | **0.48** | 0.02 | 0.14 | 0.1 |
| Family-centeredness | H1 | -0.04 | -0.02 | 0.27 | 0.24 | 0.37 | 0.13 | **0.38** | 0.04 |
|  | H2 | -0.01 | 0 | 0.17 | 0.2 | **0.33** | 0.04 | 0.23 | 0.08 |
|  | H3 | 0.04 | 0.16 | 0.24 | 0.08 | 0.23 | -0.07 | **0.36** | 0.12 |
| Community Orientation | I1 | 0.05 | 0.31 | 0.07 | -0.03 | 0.08 | 0.02 | **0.37** | -0.01 |
|  | I2 | 0 | 0.08 | -0.01 | 0.03 | 0.03 | 0.08 | **0.54** | 0.13 |
|  | I3 | -0.04 | 0.01 | 0.13 | 0.12 | 0.13 | 0.08 | **0.65** | 0.13 |
| Culturally Competent | J1 | 0.07 | 0.17 | **0.41** | 0.1 | 0.27 | 0.02 | 0.19 | 0.34 |
|  | J2 | 0.12 | 0.04 | 0.11 | 0.05 | 0.15 | 0.02 | 0.12 | **0.76** |
|  | J3 | 0.05 | 0.07 | 0.13 | 0.07 | 0.06 | -0.01 | 0.27 | **0.45** |

Exploratory factor analysis (EFA) using varimax rotation. The highest loadings for each item are bolded. Items were considered to load onto a specific factor if the factor loading was >0.40 for that factor and <0.40 for all other factors. Items are presented using wording from the administered survey.
