# Supplementary material for: Psychometric properties of the Adult Primary Care Assessment Tool Short form (PCAT-S) among high-risk patients in Australian general practice
Source: PLoS One. 2026 Feb 6;21(2):e0341250. doi: 10.1371/journal.pone.0341250 (PMC12880635; doi:10.1371/journal.pone.0341250)
Supplement: Attachment — Submitted filename: Response to Reviewers.docx [file pone.0341250.s011.docx]

**Filename: Response to Reviewers**

**Title:** Response letter for PONE-D-24-43966: “*Psychometric properties of the Adult Primary Care Assessment Tool Short Form (PCAT-S) among high-risk patients in Australian general practice”*

This document provides authors’ responses to editorial and reviewer comments. Authors’ responses are highlighted in red text. Page and line numbers are drawn from the manuscript version without tracked changes.

Part I: Response to editorial comments

1. Please ensure that your manuscript meets PLOS ONE's style requirements, including those for file naming. The PLOS ONE style templates can be found at

https://journals.plos.org/plosone/s/file?id=wjVg/PLOSOne_formatting_sample_main_body.pdf and 
<https://journals.plos.org/plosone/s/file?id=ba62/PLOSOne_formatting_sample_title_authors_affiliations.pdf>

Authors’ response: PLOS ONE file naming requirements were reviewed and files were renamed accordingly.

2. Please note that funding information should not appear in the Acknowledgments section or other areas of your manuscript. We will only publish funding information present in the Funding Statement section of the online submission form. Please remove any funding-related text from the manuscript.

Authors’ response: Funding-related text was removed from the Acknowledgements section.

3. Thank you for stating the following in the Competing Interests section:

“I have read the journal's policy and the authors of this manuscript have the following competing interests: Prof Grant Russell, Prof Jan Radford, and Prof Danielle Mazza have received honoraria from the RACGP for expert committee roles. The other authors have no conflicts to declare.”

Please confirm that this does not alter your adherence to all PLOS ONE policies on sharing data and materials, by including the following statement: "This does not alter our adherence to  PLOS ONE policies on sharing data and materials.” (as detailed online in our guide for authors http://journals.plos.org/plosone/s/competing-interests).  If there are restrictions on sharing of data and/or materials, please state these. Please note that we cannot proceed with consideration of your article until this information has been declared.

Please include your updated Competing Interests statement in your cover letter; we will change the online submission form on your behalf.

Authors’ response: Thank you. We have changed the Competing Interests statement in our cover letter accordingly.

4. We note that you have indicated that there are restrictions to data sharing for this study. For studies involving human research participant data or other sensitive data, we encourage authors to share de-identified or anonymized data. However, when data cannot be publicly shared for ethical reasons, we allow authors to make their data sets available upon request. For information on unacceptable data access restrictions, please see http://journals.plos.org/plosone/s/data-availability#loc-unacceptable-data-access-restrictions.

Before we proceed with your manuscript, please address the following prompts:

a) If there are ethical or legal restrictions on sharing a de-identified data set, please explain them in detail (e.g., data contain potentially identifying or sensitive patient information, data are owned by a third-party organization, etc.) and who has imposed them (e.g., a Research Ethics Committee or Institutional Review Board, etc.). Please also provide contact information for a data access committee, ethics committee, or other institutional body to which data requests may be sent.

**Authors’ response: Data can be made available upon reasonable request to the authors and subject to appropriate ethics approvals from the** UOW Health and Medical Human Research Ethics Committee (uow-humanethics@uow.edu.au)

b) If there are no restrictions, please upload the minimal anonymized data set necessary to replicate your study findings to a stable, public repository and provide us with the relevant URLs, DOIs, or accession numbers. Please see http://www.bmj.com/content/340/bmj.c181.long for guidelines on how to de-identify and prepare clinical data for publication. For a list of recommended repositories, please see https://journals.plos.org/plosone/s/recommended-repositories. You also have the option of uploading the data as Supporting Information files, but we would recommend depositing data directly to a data repository if possible.

Please update your Data Availability statement in the submission form accordingly.

5. In this instance it seems there may be acceptable restrictions in place that prevent the public sharing of your minimal data. However, in line with our goal of ensuring long-term data availability to all interested researchers, PLOS’ Data Policy states that authors cannot be the sole named individuals responsible for ensuring data access (http://journals.plos.org/plosone/s/data-availability#loc-acceptable-data-sharing-methods).

Data requests to a non-author institutional point of contact, such as a data access or ethics committee, helps guarantee long term stability and availability of data. Providing interested researchers with a durable point of contact ensures data will be accessible even if an author changes email addresses, institutions, or becomes unavailable to answer requests.

Before we proceed with your manuscript, please also provide non-author contact information (phone/email/hyperlink) for a data access committee, ethics committee, or other institutional body to which data requests may be sent. If no institutional body is available to respond to requests for your minimal data, please consider if there any institutional representatives who did not collaborate in the study, and are not listed as authors on the manuscript, who would be able to hold the data and respond to external requests for data access? If so, please provide their contact information (i.e., email address). Please also provide details on how you will ensure persistent or long-term data storage and availability.

**Authors’ response:** Requests for data may be made to the authors, or if unavailable, through the UOW Health and Medical Human Research Ethics Committee (uow-humanethics@uow.edu.au).

Part II: Response to Reviewer#1 comments

Reviewer #1: This paper describes the psychometric properties of an instrument that measures patients experience with primary care. While the paper was interesting to read, I do have some questions and suggestions for improvements. In the introduction several psychometric properties from other studies are reported following the aim of the study. The last sentence gives a rationale for the study. I would recommend moving these reports from other studies to the method section and allowing the introduction to end in an aim.

**Authors’ response:** Thank you for the suggestion. We agree that this is a useful change to our manuscript and have now moved reports from other studies to the Method section, and the Introduction now ends in an aim.

Reviewer #1: In the introduction I would rather see more information on the PCAT in general, when, how, and why was it developed for instance. It is described that it is one of the most widely used tools, that sounds great, but again, as a reader I need more information to be able to understand why and how this tool is useful.

From this paper it is unclear where I can find information on the development and validation of the original PCAT-scale, and its short form. This should be clearly stated with a reference when the scale is first introduced, and again in the method section, making it possible for us reviewers and later also readers to read the original study, in order to understand the theory behind the instrument, how items have been selected into the final version, if it has been analyzed for convergent and discriminant validity and so on.

Authors’ response: More information on the PCAT in general was added to the Introduction, including some statements about why the tool is useful. We added additional references to the first research papers around the development and validation of the original PCAT-scale and its short form.

Lines 52 to 71: “*The Primary Care Assessment Tool (PCAT) is widely used for evaluating primary care services globally, was developed by the Johns Hopkins Primary Care Policy Centre [4]. The PCAT has versions that are intended for consumers, including children, and health care providers. and is designed to assess the extent and quality of primary care services in provider settings identified by consumers as their predominant source of health care.*

*The PCAT is based on a theoretical model of primary care formulated by Starfield [5], which incorporates the following four essential primary care attributes: first-contact accessibility and use, continuity, comprehensiveness, and coordination, as well as family-centeredness, community orientation, and cultural competence.*

*The development of the PCAT began with the Children and Adolescents version, which was initially validated in the United States by Cassady et al. (2000) [6]. This was followed by the development and validation of the Adult Edition and the Adult Short Form, with further validation and refinement documented by Shi et al. (2001) [7].*

*The PCAT has since been translated, validated, and utilized across many countries, including Canada, Brazil, Malta, Spain, South Korea, Japan, China, Hong Kong, Taiwan, Tibet, Vietnam, Malawi, Uganda, and South Africa [4–8]. As noted by Rocha and colleagues [8], while many tools assess specific dimensions of primary care quality, there are few like the PCAT that enable a comprehensive evaluation of primary care from the population perspective. Additionally, the Consumer/Client Surveys are designed for self-administration and require only a high school reading level, making the tool broadly accessible to the general population [7].* ”

Reviewer #1: Data analysis, when describing the test-retest reliability the retesting interval is much wider than the usually recommended 2 weeks. If this method is intended to indicate temporal stability, this needs to be more clearly stated. Why should assessments one year apart show such stability? Either remove these analyses or give justification, if possible. In the discussion it gets even more confusing, when authors try to describe why temporal stability was low. The idea of test-retest is that the latent variable should be stable over the time frame for reassessment, to enable conclusions on variability or stability of results.

Authors’ response: Thank you. This is a valid query of our study design, as a one-year interval is relatively long for measuring test-retest reliability. We have provided some additional justification for using this interval:

Lines 223 to 229: “*Although test-retest reliability is typically assessed over short intervals, typically one to two weeks, to minimize the influence of external changes, longer intervals can still provide valuable insights in the context of longitudinal research. This is relevant for the PCAT, which has been used in longitudinal studies, and in measuring national and state-level primary care experiences over time in Brazil and Canada [13, 42-45]. Instruments that consistently reproduce the same result across extended periods are considered to have high temporal stability, which can be useful for longitudinal research.”*

Reviewer #1: I think the paper would benefit from adding an analysis of item-scale correlations, to discern both convergent and discriminant validity. Perhaps this would make the inconclusive results from the factor analyses clearer and add important information on the subscales.

Authors’ response: We thank the reviewer for the suggestion regarding item–scale correlations. While we agree that such an analysis could provide additional insights, we have opted not to include it in the current manuscript to maintain focus and clarity, given the already substantial length and number of analyses presented. We have, however, acknowledged this as a limitation in the manuscript and noted it as an area for future research.

Lines 484 to 486: “*We also acknowledge that the study did not assess convergent and discriminant validity, which could have been helpful for clarifying the results of the factor analyses.”*

Reviewer #1: In the result section, page 24, this CFA did show acceptable model fit indices?

Authors’ response: Yes, this is correct, CFA fit indices fell within acceptable range for the neutral-value imputation sample and EFA is not indicated in this situation. However, two of these fit indices were only borderline fit (CFI = 0.90, RMSEA 0.06, SRMR 0.07; compared to benchmarks CFI ≥0.90, SRMR <0.08, and RMSEA ≤0.06). The hypothesized model remains plausible but may benefit from further refinement. We conducted an EFA as an additional exploratory step to assess whether the data might indicate a more appropriate factor structure, and to provide a comparison across the three imputation samples.

Lines 319 to 320: “*CFA fit indices fell within an acceptable range; however, two of the fit indices indicated only borderline fit, and we therefore still conducted an EFA to further investigate factor structure*.”

Reviewer #1: How does the results from these CFA compare to other studies? One subscale has inadequate number of items for CFA, how have other studies on this scale described this limitation? How can this have impacted the results? Should the analyses be adjusted in some way?

Authors’ response: In studies which performed CFA for psychometric validation, best fitting models demonstrated goodness-of-fit statistics well within acceptable ranges – for example Berra et al 2011, Kijima et al 2021 and Wang et al 2014. However, these CFA models evaluated country-specific adaptations of the PCAT, rather than the PCAT in it’s original form. In our study, the version of the tool we examined was not an Australian adaption of the PCAT – rather, it was a version that was pragmatically modified for use in a larger RCT and these modifications were not guided by formal psychometric methods.

The subscale in question is *First-Contact Utilization,* which contains three items in the original PCAT Adult Short Form. One item was omitted for the purpose of the larger RCT (from which our study data is drawn from – see **Table S1**). However, we chose to retain all subscales in the presentation of our results, including First-Contact Utilisation, because our primary aim was to explore how an existing, previously validated instrument performs in a new context, rather than conduct a comprehensive validation of a new instrument. We recognise that the reduced item count may have contributed to the poorer model fit observed for this subscale and may have affected the overall CFA model. However, adjusting the model by removing the subscale would deviate from our intent to assess the tool as used in the RCT.

We have revised our Discussion accordingly:

Lines 491 to 506: “*One subscale (First-Contact Utilisation) included only two items – while this subscale contained three items in the original PCAT-S, one item was omitted for the purpose of the larger RCT from which our study data is drawn from. We acknowledge that while we retained it to reflect how the instrument was used in practice, this may have contributed to poorer model fit and may have affected the overall CFA model.*

*We note that in studies which performed CFA for psychometric validation of country-specific adaptations of the PCAT, best fitting models demonstrated goodness-of-fit statistics well within acceptable ranges [24, 48, 49]. Country-specific adaptations of the PCAT involve modifications to survey questions, rearranging of items into different subscales or the addition or removal of items [8, 25, 37, 39, 49, 56]. In our study, the version of the tool we examined was not a country-specific adaption of the PCAT – rather, it was a version that was pragmatically modified for use in a larger RCT and these modifications were not guided by formal psychometric methods such as content validation assessments. Our findings—that certain subscales require revision to improve reliability and validity—suggest a need to develop an Australian version of the PCAT-S. This is consistent with the broader PCAT literature, which highlights the importance of country-specific adaptation to ensure alignment with local healthcare experiences.”*

Reviewer #1: Moreover, when reading the reference to the Vietnamese version, the letters describing each subscale differs, how come? For instance, culturally competent has the letter “J” in this paper, but “K” in the Vietnamese version, making comparisons confusing.

**Authors’ response:** We agree with the reviewer that this discrepancy is confusing. The Vietnamese version is based on the PCAT Adult Expanded version, while our analysis used the PCAT Adult Short Form and additionally excludes two subscales: Coordination (Information Systems) and Comprehensiveness (Services Available) (see Supplementary Table S1). As a result, the alphabetical ordering of the subscales differs between the two versions. To clarify this for readers, we have added a note in the Methods section.

Lines 124 to 127: “*Note the alphabetical ordering of the subscales from the original PCAT-S has not been retained due to the exclusion of two subscales—Coordination (Information Systems) and Comprehensiveness (Services Available) (see Supplementary Table S1).*”

Reviewer #1: I found the conclusion of this paper confusing. As the title suggest, this paper will describe the psychometric properties of the PCAT-S in a specified sample. Then the conclusion is that findings were mixed, and more research is needed, however the scale could be used in its current version in Australia. With reliability levels below recommended values, how would that impact interpretations of results?

Authors’ response: Thank you for this helpful feedback. We agree that our conclusion required clarification and have revised this section of the manuscript (see below).

Lines 517 to 524: “*At present, there are no validated instruments specifically designed for evaluating primary care patient experiences in Australia (except for PAIS and PESS, which focus on different domains [61]), reinforcing the PCAT's value as a tool for this purpose. This highlights the value of the PCAT as a potential tool for this purpose. Our initial validation of the PCAT-S in an Australian general practice sample showed mixed psychometric performance. While some subscales demonstrated acceptable reliability and model fit, others fell below recommended reliability thresholds. Hence survey results should be interpreted with nuance, and consideration of item relevance and interpretation in the Australian context.*

Reviewer #1: To the best of authors knowledge from reading validation studies from other countries, combined with the results from the current study, what are the next steps? From these results, what adaptations do you suggest, and how should these be addressed in future research?

Authors’ response: We have added to the conclusion to outline the next steps for research and instrument development. This includes specific recommendations for item refinement, methodology, and psychometric testing.

Lines 525 to 539: “*Next steps should include the development of a country-specific Australian version of the PCAT. As with other country-specific adaptations of the PCAT, item refinement and testing should be guided by formal face and content validity assessments, expert consultation, and engagement with patients and providers . Item refinement should include reviewing relevance of items which may not be relevant to how general practice is currently delivered in Australia (such as after-hours care, home visits, general practice involvement in community health services, discussion of home safety, and facility-related conflict). Consideration should also be given to how provider and specialist services operate within the Australian healthcare system. Instrument development should use data collected specifically for psychometric analysis and aim to sample more widely across demographic groups. More advanced psychometric testing, including assessments of convergent and discriminant validity and exploration of alternative factor structures, would further support the development of a robust and contextually appropriate instrument.*

*A validated version of the PCAT-S tailored to the Australian context would enhance the ability to measure and compare primary care performance both nationally and internationally, and support ongoing improvement in patient care.”*

Part III: Response to Reviewer#2 comments

Reviewer #2: I appreciate the authors’ providing a rationale for using the neutral-value imputation scheme, bolstered by citing multiple prior publications that used this method (lines 188-191). Presenting three sets of results adds complexity that may not be of interest to all readers. Perhaps some information could be transferred to supplemental tables.

Authors’ response: Thank you for the suggestion. We have now transferred some information to supplemental tables (Tables 3 to 6 in the original manuscript are now **Tables S6 to S9** in the revised manuscript).

Reviewer #2: The rationale for conducting EFA could be strengthened. The authors have already established the factor structure varies across different national health care contexts, based on the citations provided and the discussion on lines 94-104. If we already know the factor structure is not invariant across nations, do we need further evidence of this? This rationale could be clarified.

Authors’ response: This is a valid concern from the reviewer regarding the necessity of performing an EFA. In our manuscript, we stated the rationale for conducting the EFA was to further examine the factor structure, given that data did not fit our initial CFA model. We agree that our rationale could be strengthened and provide the following additional statements:

*Lines 172 to 176: “Since the data did not fit the hypothesised CFA model [7], an additional exploratory factor analysis (EFA) was conducted. Conducting an EFA following CFA is useful for exploring and refining the factor structure when a CFA model does not adequately capture the underlying data structure. EFA can help to identify modifications (e.g. suggesting which items load better on which factors) or provide alternative configurations that may improve model fit.”*

Reviewer #2: Would it be possible to report what proportion of patients met inclusion criteria by virtue of their age alone (65 years or older) versus being in the 18-64 age group and having a chronic illness? This might be helpful when interpreting the mean (SD) age in Table 1; 66.9 years seems somewhat low and could indicate a substantial portion of the sample were younger than 65.

Authors’ response: An age group breakdown has been added to Table 1 in the Results section of the report:

| Age (years) | |
| --- | --- |
| 18–64 years | 263 (36.8) |
| 65 years and older | 451 (63.1) |

Reviewer #2: It would be useful to more closely examine the data to identify causes for poor model fit (especially in the CFA). The sample size is inadequate for the model with no imputation but adequate for both imputation models, so isn’t a likely culprit. However, multicollinearity statistics would be helpful for the reader (and could be included in the supplemental materials). Reducing the number of factors for the EFA could also improve model fit.

Authors’ response: Thank you for the suggestion. Multicollinearity statistics have been added to our manuscript in **Table S2**. Potential multicollinearity was identified in the non-imputed sample, but not for either of the imputed datasets.

Lines 162 to 163: “*Multicollinearity risk was assessed by visualising correlation matrix and examining for small determinant of the correlation matrix (< 0.00001) – results are presented in Table S2 [30].*”

Lines 273 to 274 “*The determinant of the correlation matrix was close to 0.0001 (Table S2), suggesting potential multicollinearity in the non-imputed sample.”*

Reviewer #2: The list of exceptions seems rather lengthy when stating the factor structure of the EFA (e.g., lines 263-267). Beginning by pointing out five items (of 28) did not load as expected would frame things differently. The authors’ existing language is correct, but it caused me to mark the margin with, “that’s an awful lot of exceptions!”

Authors’ response: Yes, we agree, and have edited our manuscript accordingly.

*Line 288: “The following deviations from the expected factor structure were observed…”*

Reviewer #2: Including only participants in the control arm in the test-retest analysis is entirely appropriate. Is it possible to state the mean (median, range) length of time elapsed between the two measurement points? This may have been reported elsewhere but it’s key to interpreting the test-retest results.

Authors’ response: Yes we agree, and have added this information to the Results section of the manuscript.

*Lines 220 to 223 “* *As previously described [13, 14], the active intervention period spanned 12 months (1 August 2018 to 31 July 2019). Among control group participants, the interval between baseline and follow-up assessments ranged from 100 to 372 days, with a mean of 311 days and a median of 313.5 days.”*

Reviewer #2: The authors state reducing the number of factors is beyond the scope of this study—fair enough. However, the rationale was that changing the number of factors would be more appropriate for “face and content validity assessments” (line 468). Contrast this with the Discussion section, which seems to discuss the face validity of numerous individual items. (The term ‘face validity’ is not used, but that seems to be what’s happening.) Some of this discussion seems to go beyond the data. Rewording or reframing could resolve this apparent discrepancy.

Authors’ response: Thank you. This is an important point that the reviewer has raised. Throughout our discussion, we provide insights and contextual interpretations that we believe may help in understanding the results of the study. We have revised the introductory paragraph of our discussion to clarify that while we did not conduct formal face validity assessments, we attempted to relate our findings to contextual factors, which we hope provide some useful insights for readers and research directions.

*Lines 361 to 363: “Readers should note that we did not conduct formal face validity assessments. However, we attempted to relate our findings to contextual factors, which we believe have provided useful insights for interpreting the results and potential areas for future research.”*

Reviewer #2: The authors reference “poor temporal stability (α = 0.32).” This value is the ICC reported in Table 7. Would it be clearer to use rho in place of alpha, here?

Authors’ response: We thank the reviewer for pointing out this error. This has been corrected in the manuscript.

Reviewer #2: Is it possible to rule out having changed one’s GP as an explanation for the low test-retest reliability of the “First contact – Utilization” subscale? The authors posit changing providers as a potential explanation (lines 357-361). It’s possible the larger RCT has data on whether a participant switched practices. Did participants in the control arm have a shorter duration of relationship with the GP (or practice) than those in the treatment arm at baseline? Given that more than three-quarters of participants had been with their GP (or practice) over 5 years, changing providers doesn’t seem an especially compelling explanation for the low ICC on this subscale.

Authors’ response: The reviewer provides insightful and valid arguments regarding the improbability of changing one’s GP as an explanation for the findings in this particular study cohort. Unfortunately, a ***Change in GP*** variable was not included in the original design of the larger RCT.

Regarding the low ICC score, we believe the low test-retest reliability is primarily due to statistical limitations, including the large ceiling effect observed in both items of the subscale, as well as the subscale’s limited item count (only two). These factors constrain response variability and are more likely contributing to the unexpectedly low ICC score, than changing providers

We have replaced the sentences in our Discussion accordingly *(Lines 371 to 373).*

*Removed: “A low value of alpha could be due to a low number of questions, factors such as poor inter-relatedness between items or heterogeneous constructs [38]. Additionally, the instability may reflect changes in provider use, as participants who switch GPs or practices could naturally change their responses to these items.”*

*Replaced with: “This unexpectedly low test-retest reliability is most likely due to statistical limitations, including the large ceiling effect observed in both items of the subscale and the subscale’s limited item count (only two)*”.
