# Supplementary material for: Psychometric properties of the Adult Primary Care Assessment Tool Short form (PCAT-S) among high-risk patients in Australian general practice
Source: PLoS One. 2026 Feb 6;21(2):e0341250. doi: 10.1371/journal.pone.0341250 (PMC12880635; doi:10.1371/journal.pone.0341250)
Supplement: Attachment — Submitted filename: 22Nov2025-Response to reviewers.docx [file pone.0341250.s012.docx]

21 November 2025

**Editorial Board**

*PLOS ONE*

Dear Editors,

We have carefully revised the manuscript in response to the feedback provided. Below, we provide a response to each reviewer comment, with our responses shown in red text.

As recommended by the reviewers, we have removed the test–retest analyses from the manuscript. This resulted in corresponding changes to the Reference List, which we note here in accordance with the Journal Requirements.

Thank you for your consideration of our manuscript. We look forward to your response.

Sincerely,

Dr Chau Bui, PhD

Graduate School of Medicine

University of Wollongong Australia

Reviewer #1: 

I do agree with the authors that temporal stability is important for longitudinal research. However, I am still not convinced that these analyses address test-retest reliability. The idea with test-retest analysis is that the latent variable is assumed to be stable between measurement occasions, thus differences found between measurements indicate errors in the scale. High test-retest reliability indicates that these errors are negligible. In the present study, why is it assumed that the latent variable should be stable? In longitudinal research, sensitivity to change is perhaps even more important than temporal stability. Could these results imply that the instrument is sensitive enough to detect changes?

**Author’s response**. We thank the reviewers for their comments regarding test-retest reliability. After careful consideration, we have removed the test-retest analyses from the manuscript. We agree that in longitudinal research, the ability of an instrument to detect real changes over time can be more relevant than assuming complete stability of the underlying construct. In our study, the one-year interval between measurements means that observed differences may reflect true changes in participants’ experiences rather than measurement error. While we had previously justified that using a long interval can provide information on temporal stability, this is only in a general sense, and they cannot be interpreted as classical test-retest reliability, which assumes the underlying construct remains stable.

When looking at table 2, it seems there are high proportions of responses in the highest possible score. The set ceiling effect of above 80% is quite high, how did authors arrive at this level for indicating ceiling effect? Authors have made it clear how this could have impacted the results, and the selected estimator DWLS is appropriate for cases where ceiling effects are high, however I am unfamiliar with the above 80% rule.

**Author’s response**: We thank the reviewer for this comment. Our initial 80% threshold for defining a ceiling effect was based on a previous PCAT validation study (Hoa et al., 2018), but we recognize that this exceeds commonly recommended standards, which typically define floor or ceiling effects as present when more than 15–20% of respondents achieve the minimum or maximum score. We have revised the manuscript to use the 20% threshold, and applying this shows that all but one of the 28 items exhibit high floor or ceiling effects. The results and discussion have been updated accordingly.

Page 6, line 152: *“80%”* was changed to *“20%”*

Page 13, lines 318 to 320: *“Based on the standard 20% criterion, 5 of the 28 items showed large floor effects and 23 items showed large ceiling effects. Additionally, very high ceiling effects (>80%) were noted for the two first-contact utilization items (C1 and C2) and for item J1.*”

Page 26, lines 564 to 565: *“In addition, nearly all items exhibited floor or ceiling effects, likely reflecting the homogeneity of our sample.*”

Hoa NT, Tam NM, Peersman W, Derese A, Markuns JF. Development and validation of the Vietnamese primary care assessment tool. PLoS One. 2018;13(1):e0191181. Epub 20180111. doi: 10.1371/journal.pone.0191181. pmid: 29324851; PubMed Central PMCID: PMC5764365.

Please review the use of periods and commas throughout the text, and typos, for example:
Page 3, line 54, “… and health care providers. and is …”
Page 6, line 139: “… effects were defined as items wwhere > 80% of ...”
Page 20, line 316, “… and to provide a comparison across the three imputation samples.Results …”

**Author response:** We thank the reviewer for highlighting these issues. We have carefully proofread the final version of the manuscript to correct any typos.

Reviewer #2: The commnts from both reviewers were addressed within the revised manuscript. Three concerns remain although I recommend publication without requiring a third round of review. (Note the line numbers refer to the markup version.)

**Authors’ response:** We have added citations to support the rationale for conducting EFA following CFA. First, we cite Flora and Flake (2017, pages 20–21, Chapter “Model Modification”), which discusses moving from a poorly fitting CFA to an exploratory factor analysis to identify alternative factor structures. Second, we cite a practical example of this approach: Ryan et al. (2019) applied EFA after CFA when the hypothesized CFA model did not adequately fit their sample.

Page 8, lines 192 to 194: “ *When a hypothesized CFA model does not adequately fit the data, it is acceptable practice to use EFA to explore the underlying factor structure and identify alternative item loadings or configurations [**35**,* *36**].”*

Flora D, Flake J. The Purpose and Practice of Exploratory and Confirmatory Factor Analysis in Psychological Research: Decisions for Scale Development and Validation. Canadian Journal of Behavioural Science . 2017;49:78–88. doi:10.1037/cbs0000069.

Ryan J, Curtis R, Olds T, Edney S, Vandelanotte C, Plotnikoff R, et al. Psychometric properties of the PERMA Profiler for measuring wellbeing in Australian adults. PLoS One. 2019;14(12):e0225932. doi:10.1371/journal.pone.0225932. PMCID: PMC6927648.

2. I echo Reviewer 1's concerns regarding test-retest reliability. I appreicate few studies have evaluated this aspect of the instrument (lines 301-303). The authors note a single test-retest including only 15 respondents, which could indicate the need for further validation (test-restest reliability) of the scale. Still, even that small analysis was conducted over a two-week timeframe rather than a year. Given the low test-retest reliability for the First Contact-Utilization subscale, how can this establish temporal stabliity in a way that's useful for future longitudinal research? No one study can "do it all." If the design of the larger study was such that test-restest reliability was unable to be assessed, I suggest dropping the test-retests analyses form this manuscript.

**Author’s response:** We thank the reviewer for their thoughtful comments regarding test-retest reliability. We agree that the design of the larger RCT, which uses a one-year interval, precluded a meaningful assessment of test-retest reliability. After careful consideration, we have therefore removed the test-retest analyses from this manuscript and revised the text accordingly.

3. A careful proofreading (and perhaps professional copyediting?) is strongly recommended. Some redudant (or nearly-redundant) language was introduced by the revisions. One example of this near-reptition occurs in lines 655-656: "...reinforcing the PACT's value as a tool for this purpose. This highlights the value of the PACT as a potential tool for this purpose."

**Author’s response:** We thank the reviewer for highlighting these issues. We apologize for the oversights and have carefully proofread the final version of the manuscript to remove redundant or repetitive language.
